# Surgical Operations in Private Practice

**Published:** 1904-12-03

**Authors:** John Aikman


### Hospital Clinics.

### SURGICAL OPERATIONS IN PRIVATE PRACTICE.

By John Aikman, M.D., C.M., Glas.

### IV. The Preparation of Rooms and Instruments.

In the surgical procedures which we have noticed the surroundings have been outside our choice, as in compound fracture; or the wounds which we have made have been so small as to keep the channels of septic infection within easy control. It is not always so. In many cases, as in an operation on the abdomen, the surgeon opens up vast spaces which are ready to receive infection other than that which our hands or instruments can carry. It was to meet this infection that the antiseptic spray was designed. We learnt first that we could prevent sepsis in a wound by destroying the recent infection by antiseptics; we learnt later that the endeavour to disinfect the atmosphere by such means was not satisfactory. There was a bifurcation in our road, and for choice—

> " then, some sage acquaint
> The simple—which holds sinner, which holds saint!"

To-day, we recognise that all things may be made clean: wounds by washing and antiseptics, atmosphere by cleansing such surroundings as harbour dust. In surgical practice we recognise two kinds of dust—the first is the microbic dust of the specific infections, as when a room has been occupied by a case of zymotic disease; the second is the common dust of wear and tear, which forms a nidus for all septic and infectious organisms. In dealing with an infected room the common dust is always the more important; it should be first removed. The specific dust may then be dealt with by aerial disinfectants, and I think that sulphur and chlorine are more effectual than formalin. The exhibition of either in sufficient quantity, and for a sufficient time, will purify a room which has been freed from the nidus of common dust.

To remove the common dust is not an easy matter; it is a wily foe, but thanks to modern workers its strategy is an open book. All our more useful surroundings pass towards decay by a process of attrition; friction diminishes the cohesion of their particles, and permits the entrance of the germs which make them septic. These winged particles float in the atmosphere and render it pernicious and dangerous. The natural apertures of the body are provided with means for the entanglement, filtration, or destruction of this dust, and safeguard us; the complex course of the breath in the nostril over a moist surface of membrane is a type of entanglement, the function of the lymphatic glands is that of filtration. But it is different with surgical wounds; all the first line of defence is removed, only the action of the phagocytes remains, and that not at its best. It devolves upon the surgeon to insure that no infected dust shall reach the wound which he makes; hence the maxim: "No dust, no disease."

To prepare a room for an operation, carpets, curtains, and all hangings must be removed. The ledges over doors and windows must be cleansed and freed from the dust which is apt to rest upon them. Ceilings must be rubbed with a brush covered by a damp cloth, and floors scrubbed until they are clean and sodden. It is enough to rub the walls down; and bedsteads, gas brackets, and tables should be rubbed until they have no detachable particles left upon them. The principle is simple—to remove all particles which have become detachable, and to render aseptic what remains. Soap in a six per cent. solution is a very good antiseptic, and it has the additional advantage of causing any dust which may not have been removed to adhere to the surface which is left moist with it, for a longer time than most operations last. Scarcely less important is the cleansing of the room from all blood-stains and dressings after the operation is over, and before the patient is sufficiently recovered from the anæsthetic to be conscious of the noise. Soap and water is all that is required, but the door and window-ledges must not be forgotten, for that light and omnipresent agent, horse manure, is almost unavoidable in the air which enters the room in the ventilation which is needful during an operation, and it carries with it the heat which aids bacterial growth.

Towels and sheets which have been recently washed are fairly aseptic, and may be rendered safe by immersion in a two-and-a-half per cent. phenol solution. Linen and cotton fabrics which have been stored for any length of time should not be used.

Most instruments can be sterilised by boiling on the day of operation. I usually place the boiled instruments in a measured quantity of five per cent. phenol solution before the patient is anæsthetised, and dilute it to the needful degree when anæsthesia is complete.

The keeping of instruments is almost more important; it never does to allow blood or secretions to dry on instruments, they should be washed immediately with soap and water, and the teeth of pressure forceps and such things scrubbed with a nail-brush. No amount of boiling will render instruments reliable which have blood or discharges dried upon them. But if they have been cleansed and then boiled, they may be packed away wrapped in a fine linen cloth, and be ready to use with slight purification in cases of emergency, though the fuller preparation is better.

I have more faith in well prepared sponges than in pads of wadding; they are less apt to leave debris in the wound. Sponges may be cleaned by ether soap and washing, but the process takes about eight days and requires much attention.

The soap is not needful after the first few days, but the water must be changed frequently, and it should always be boiled water. Once cleansed, the sponges should be kept in five per cent. phenol solution, but the phenol is apt to crystallise in the pores of the sponge which should be well soaked in boiling water before being used.

Stitch abscess is one of the puzzles of the surgeon's life; it does not occur under circumstances which would seem to favour it, and it does occur when every precaution appears to have been observed. Silkworm gut ligatures are the most immune, and I have attributed this to their smooth surface; catgut and silk are more apt to fray and I have become accustomed to test them by friction on a sponge or sterile cloth before use. The accident is too rare to give certain results from a personal experience, but this seems worthy of a note.

Knives are always a difficulty; the edge is spoiled by boiling, and although a keen edge holds little dust, a knife which has been used is less likely to be pure. Liquified phenol acts like the alcohol, which it is, and cleanses well, if the edge is smooth and keen; but used knives should be boiled. A new knife is better for incisions in skin and membranes of close texture where it is desirable to have a very clean cut; a less keen edge will suffice for the looser structures. The differentiation corresponds to the after remodelling.

In all matters relating to the preparation of rooms and instruments the great safeguard is vigilance, mixed, not with formulæ, but with "brains."

